# Thrombus migration in intracranial large vessel occlusion: course, predictors, and impact on endovascular thrombectomy

**DOI:** 10.3389/fneur.2025.1647008

**Published:** 2025-09-19

**Authors:** Mi-Yeon Eun, Woochan Choi, Yang-Ha Hwang, Kwang Hyun Kim, Yong-Won Kim

**Affiliations:** ^1^Department of Neurology, School of Medicine, Kyungpook National University, Daegu, Republic of Korea; ^2^Department of Neurology, Kyungpook National University Chilgok Hospital, Daegu, Republic of Korea; ^3^Department of Neurology, Graduate School, Korea University, Seoul, Republic of Korea; ^4^Department of Neurology, Kyungpook National University Hospital, Daegu, Republic of Korea

**Keywords:** thrombus migration, ischemic stroke, large vessel occlusion, endovascular thrombectomy, thrombus composition

## Abstract

**Introduction:**

Thrombus migration (TM) is occasionally observed in patients with acute ischemic stroke undergoing endovascular thrombectomy (EVT) for large vessel occlusion. However, the predictors and clinical implications of TM remain unclear. This study aimed to identify clinical and radiological factors associated with TM and assess its impact on procedural and functional outcomes.

**Materials and methods:**

We retrospectively analyzed 348 patients with intracranial large vessel occlusion (ICA, M1, or M2) treated with EVT at two comprehensive stroke centers. TM was defined as a distal shift of the thrombus location between CT angiography and digital subtraction angiography. Predictors of TM were determined using multivariable logistic regression. Procedural and clinical outcomes were compared between the TM and non-TM groups.

**Results:**

TM was observed in 77 patients (22.1%), with 32 patients showing migration beyond the vessel segment. In the multivariable analysis, hyperdense artery sign [HAS; adjusted odds ratio (OR), 4.68; 95% confidence interval (CI), 2.62–8.34], diastolic blood pressure (adjusted OR, 0.98; 95% CI, 0.96–1), and onset-to-arrival time per 60 min (adjusted OR, 0.87; 95% CI, 0.79–0.97) were associated with TM. The TM group showed greater NIHSS improvement, with a trend toward higher first-pass effect rates. Parenchymal hemorrhage was more frequent in the TM group. However, successful reperfusion and 3-month functional outcomes were comparable between groups.

**Conclusion:**

In patients with intracranial large vessel occlusion, HAS, diastolic blood pressure, and onset-to-arrival time were associated with TM. These findings suggest a role for thrombus composition in the TM. Radiologic and clinical outcomes were comparable in the TM and non-TM groups.

## Introduction

Endovascular thrombectomy (EVT) has become the standard of care for patients with acute ischemic stroke caused by large vessel occlusion. Current guidelines advocate for a combined approach of EVT with best medical management, such as intravenous thrombolysis (IVT), specifically for large vessel occlusions in the anterior circulation (1–3).

The location of arterial occlusion, influenced by the thrombus origin and individual vascular anatomy, plays a critical role in the decision to perform EVT and affects clinical outcomes. Thrombus migration (TM), defined as the distal movement of thrombus, is occasionally observed in patients with large vessel occlusion and has been increasingly recognized with the widespread use of EVT. However, its clinical relevance and predictive factors remain unclear. TM may result from spontaneous displacement or partial thrombus resolution, especially after IVT. Considering this mechanism, factors known to predict recanalization after IVT in large vessel occlusion, such as thrombus length, composition, and time to treatment (4–6), may influence the likelihood of TM. Previous studies have suggested that IVT (3, 7) and thrombus burden (8) may be associated with the occurrence of TM. Additionally, TM may be influenced by various hemodynamic factors, collateral status, or premorbid antithrombotic treatment.

Despite these insights, few studies have comprehensively evaluated the combined effect of these variables on TM. We hypothesized that thrombus characteristics and time parameters may be the key factors for TM. Therefore, we aimed to investigate the natural course and independent predictors of TM and their impact on outcomes in patients undergoing EVT through detailed clinical and radiological evaluation.

## Materials and methods

### Study design and participants

We conducted a retrospective analysis using data from a prospective, hospital-based stroke registry at two tertiary stroke centers between January 2017 and December 2022. The registry included all consecutive patients with acute ischemic stroke within 7 days of onset and consisted of demographic data, medical history, stroke severity and etiology, and clinical outcomes. Patients were eligible if they had an acute ischemic stroke due to intracranial large vessel occlusion in the anterior circulation and underwent EVT. The inclusion criteria were as follows: (1) patients aged 19 years and older and (2) patients with acute ischemic stroke undergoing EVT for the acute occlusion of the intracranial internal carotid artery (ICA), middle cerebral artery (MCA) M1 or M2 time from symptom onset to arrival within 24 h. Patients were excluded if they did not have a computed tomography (CT) scan of the brain and CT angiography within 6 h before the procedure. Patients with multivessel occlusion, such as bilateral MCA occlusion or MCA and anterior cerebral artery occlusion, were also excluded. The Institutional Review Board of each hospital approved this study. Informed consent was waived due to the retrospective nature of the study and anonymized data analysis.

### Clinical data and imaging assessment

Clinical and imaging data were obtained from electronic medical records and the hospital-based stroke registry. Information collected included demographics, vascular risk factors, medication history, etiologies based on the Trial of Org 10,172 in Acute Stroke Treatment (TOAST) classification (9) and the use of IVT, EVT modality, and rescue therapy. Time metrics were recorded, including time from symptom onset to arrival at the emergency department, from arrival to imaging, from arrival to IVT, from imaging to arterial puncture, and from puncture to recanalization.

The Alberta Stroke Program Early CT Score (ASPECTS) was assessed on non-contrast brain CT to determine the extent of early ischemic changes (10). To evaluate the characteristics of the thrombus that influence TM, we assessed the presence of the hyperdense artery sign (HAS) in the occluded artery on non-contrast brain CT. HAS was defined as any increased density of the intracranial arterial lumen on non-contrast CT compared with adjacent or equivalent contralateral arteries on predefined brain CT settings (window width 80, window level 40) with adjustment (11, 12). We also evaluated the clot burden score (CBS) to assess the extent of the intracranial thrombus, which ranges from 0 to 10, with a lower score indicating a larger clot (13). Baseline collaterals were assessed on CT angiography and classified into good (2, 3) and poor (0–1) (14).

### Assessment of thrombus migration

**Figure 1:**
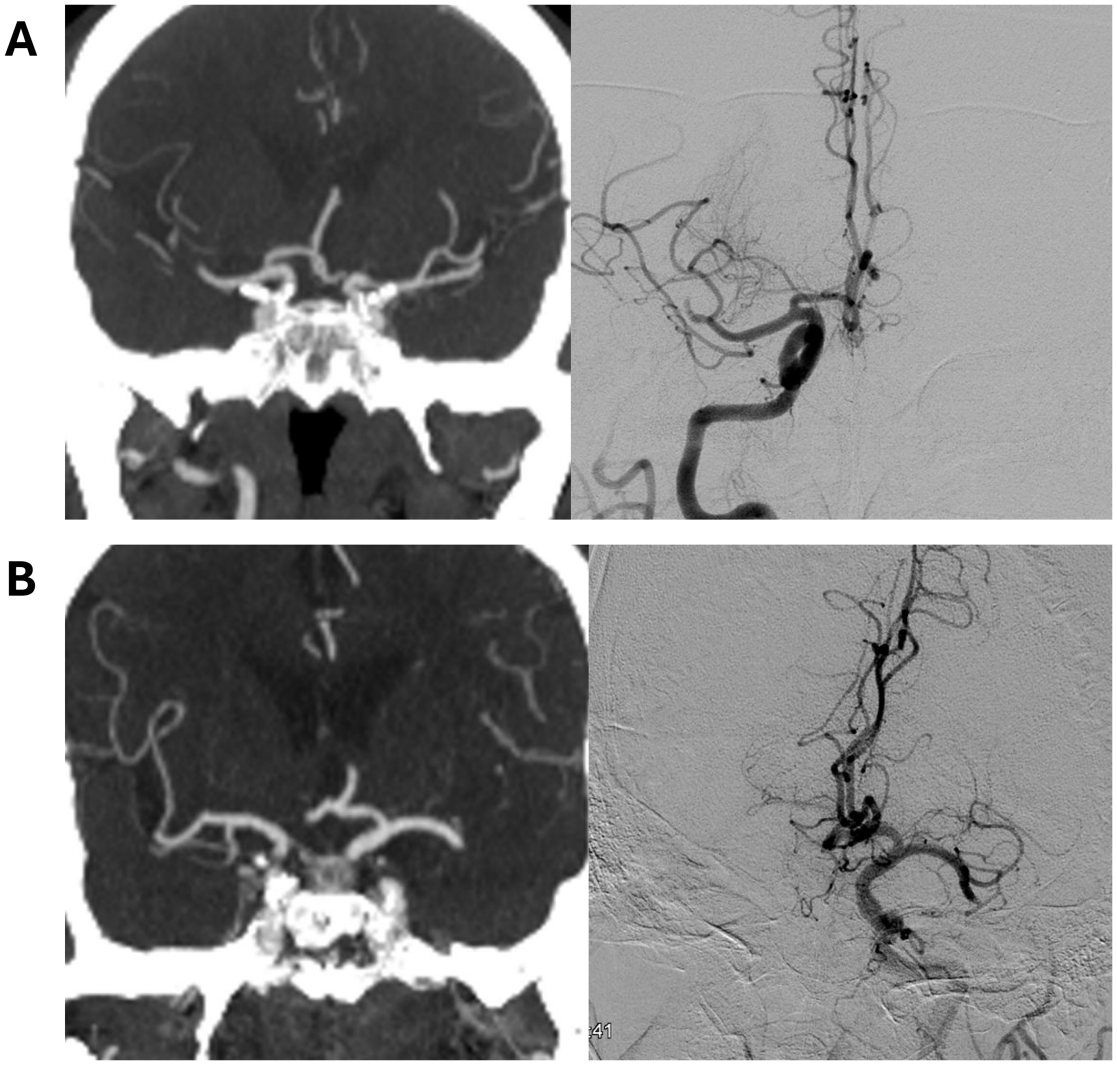
Definition of thrombus migration by discrepancy between CT angiography and digital subtraction angiography. Representative examples show **(A)** intersegmental thrombus migration from the right MCA M1 segment to the M2 segment and **(B)** intrasegmental thrombus migration in the left MCA.

Thrombus location was assessed on pre-interventional CT angiography and digital subtraction angiography (DSA) before EVT. CT angiography included both single-phase and multiphase acquisitions. For multiphase studies, thrombus location and length were evaluated on the arterial phase, which most closely corresponds to the single-phase acquisition, to ensure consistency across patients. TM was defined as any distal movement of the proximal end of the thrombus on DSA compared with CT angiography (Figure 1) (3). Thrombus growth was not classified as TM. Brain imaging was independently reviewed by two interventional neurologists (Eun M.-Y. and Choi W.-C.) who were blinded to the clinical and radiological outcomes, and any disagreements were resolved by discussion and confirmed by the senior neurointerventionalist (Kim Y.-W.).

### Endovascular thrombectomy

We performed EVT in patients with acute stroke who had large vessel occlusion concordant with the international (1) and Korean guidelines (15) and our institutional standard operating procedure. Briefly, patients underwent EVT if they presented within 6 h of symptom onset or between 6 and 24 h with evidence of a clinical-imaging mismatch or a mismatch between the infarct core and penumbra on imaging.

Patients eligible for IVT were treated with intravenous alteplase before EVT. All patients underwent EVT under local anesthesia. Either stent retriever or contact aspiration thrombectomy was performed as a first-line modality at the discretion of the treating neurointerventionalist.

### Clinical and radiological outcomes

We performed clinical assessment using the National Institutes of Health Stroke Scale (NIHSS) score on the day of admission, at 24 h after the EVT, and at 7 days after admission. We calculated the delta NIHSS by subtracting the baseline NIHSS from the NIHSS at 24 h after EVT, and early neurological improvement (ENI) was defined as a delta NIHSS score of at least 8 points or achieving a score of 0–1 points at 24 h after EVT (16). Functional outcome was measured with a modified Rankin Scale (mRS) score at 3 months in the outpatient clinic or by structured telephone interviews by a certified neurologist or trained nurses. A good outcome was defined as mRS 0–2 at 3 months.

Reperfusion status was assessed on the final DSA and graded using the modified Thrombolysis in Cerebral Infarction (mTICI) scale. Successful reperfusion was defined as mTICI 2b or 3 (17). We also assessed first pass effect (FPE) and modified FPE (18). Intracranial hemorrhage (ICH) was assessed on the non-contrast CT immediately and 24 h after EVT and classified according to the European Cooperative Acute Stroke Study (ECASS) III definition (19). Symptomatic ICH was defined as any ICH, such as intracerebral hemorrhage, subarachnoid hemorrhage (SAH), and intraventricular hemorrhage, associated with an increase in an NIHSS score of 4 or more. Distal embolization to the distal territory or new territories during EVT was also assessed.

### Statistical analysis

Continuous variables were presented as mean ± standard deviation or median and interquartile range (IQR), as appropriate. Categorical values were presented as frequencies and percentages. For continuous variables with less than 5% missing values in demographics and time variables, the median values were imputed. Participants were categorized into TM and no-TM groups. We compared the demographics and clinical and radiological outcomes between groups using *χ*^2^ test or the Fisher exact test for categorical variables and the *t*-test and Student’s *t*-test or Wilcoxon rank-sum test for continuous variables, as appropriate.

Univariate and multivariable logistic regression analyses were conducted to identify independent predictors for TM and to evaluate the effects of TM on clinical and radiological outcomes. Time parameters were included in the logistic regression per 60-min increments to obtain clinically meaningful odds ratios. Variables with *p*-values <0.1 in the univariate logistic regression analysis and IVT (3), previously reported to be associated with TM, were included in the multivariable analyses. A two-sided *p*-value of <0.05 was considered statistically significant. Data analyses were performed using R software (version 4.3.2; r-project.org).

## Results

### Baseline characteristics

Of the 411 patients who received EVT for anterior circulation large vessel occlusion within 24 h of symptom onset, 348 were included in the final analysis. We excluded 63 patients: 41 without pre-procedural CT or CT angiography, 6 with multiple large vessel occlusions, and 16 with indeterminate TM status. The median age was 73 (IQR, 64–80) years, and 187 (53.7%) patients were men. The median NIHSS score was 16 (IQR, 11–20), and the median ASPECTS score was 9 (IQR, 7–10). Demographics and vascular risk factors were similar between the TM and no-TM groups, except for significantly lower diastolic blood pressure in the TM group. Regarding temporal factors, patients in the TM group had a significantly shorter time from symptom onset to hospital arrival [75 min (IQR, 45–202)] compared to those in the no-TM group [135 min (IQR, 55–306)]. Occlusion site, CBS, and collateral status were comparable between groups. However, the prevalence of HAS was significantly higher in the TM group (71.4%) than in the no-TM group (37.3%). Baseline characteristics according to TM status are summarized in Table 1.

**Table 1:** Baseline characteristics of the patients according to thrombus migration.

Variables	No TM (*n* = 271)	TM (*n* = 77)	*p* value
Age, median (IQR)	72 (64–79)	73 (65–82)	0.14
Male	143 (52.8)	44 (57.1)	0.582
Hypertension	161 (59.4)	46 (59.7)	>0.999
Diabetes mellitus	89 (32.8)	22 (28.6)	0.568
Dyslipidemia	132 (48.7)	42 (54.5)	0.438
Prior stroke	46 (17.0)	19 (24.7)	0.172
Atrial fibrillation	151 (55.7)	50 (64.9)	0.189
Coronary artery disease	35 (12.9)	13 (16.9)	0.482
Smoking history	61 (22.5)	18 (23.4)	0.995
Prior antiplatelet use	66 (24.4)	24 (31.2)	0.29
Prior OAC use	46 (17.0)	15 (19.5)	0.733
SBP, median (IQR)	156 (136–169)	146 (127–172)	0.078
DBP, median (IQR)	84 (73–99)	80 (72–90)	0.01
Blood glucose, median (IQR)	128 (111–160)	123 (107–156)	0.372
NIHSS, median (IQR)	16 (10–20)	15 (12–20)	0.454
Previous mRS, median (IQR)	0 (0–1)	0 (0–1)	0.757
TOAST classification			0.905
LAA	54 (19.9)	15 (19.5)	
CE	164 (60.5)	49 (63.6)	
ODE	16 (5.9)	3 (3.9)	
UDE	37 (13.7)	10 (13.0)	
Time parameters			
Onset-to-arrival, median (IQR)	135 (55–306)	75 (45–202)	0.006
Arrival-to-image, median (IQR)	19 (15–24)	18 (14–22)	0.161
Arrival-to-IVT, median (IQR)	33 (28–44)	31 (25–41)	0.258
Image-to-puncture, median (IQR)	48 (37–63)	47 (35–58)	0.539
Imaging parameters			
ASPECTS, median (IQR)	9 (7–10)	9 (8–10)	0.857
Hyperdense artery sign	101 (37.3)	55 (71.4)	<0.001
CBS, median (IQR)	6 (6–8)	6 (5–8)	0.167
Target occlusion on CTA			0.699
ICA	69 (25.5)	21 (27.3)	
M1	156 (57.6)	46 (59.7)	
M2	46 (17.0)	10 (13.0)	
Occlusion on DSA			<0.001
ICA	72 (26.6)	8 (10.4)	
M1	154 (56.8)	40 (51.9)	
M2	45 (16.6)	29 (37.7)	
Extracranial ICA occlusion	23 (8.5)	8 (10.4)	0.771
Collateral grade on CTA			0.465
0–1 (Poor)	98 (36.2)	32 (41.6)	
2–3 (Good)	174 (64.2)	45 (58.4)	
Treatment			
IVT	102 (37.6)	35 (45.5)	0.268
EVT modality			0.425
Contact aspiration	248 (91.5)	67 (87.0)	
Stent retrieval	22 (8.1)	10 (13.0)	
Glycoprotein IIb/IIIa Inhibitor	32 (11.8)	1 (1.3)	0.011
Number of pass, median (IQR)	2 (1–3)	2 (1–3)	0.178

TM, thrombus migration; IQR, interquartile range; OAC, oral anticoagulants; SBP, systolic blood pressure; DBP, diastolic blood pressure; NIHSS, National Institutes of Health Stroke Scale; mRS, modified Rankin Scale; TOAST, Trial of org 10,172 in acute stroke treatment; LAA, large artery atherosclerosis; CE, cardioembolism; ODE, other determined etiology; UDE, undetermined etiology; IVT, intravenous thrombolysis; EVT, endovascular thrombectomy; ASPECTS, Alberta Stroke Program Early CT Score; CBS, clot burden score; CTA, computed tomography angiography; DSA, digital subtraction angiography; ICA, internal carotid artery.

### Distribution of thrombus migration

**Figure 2:**
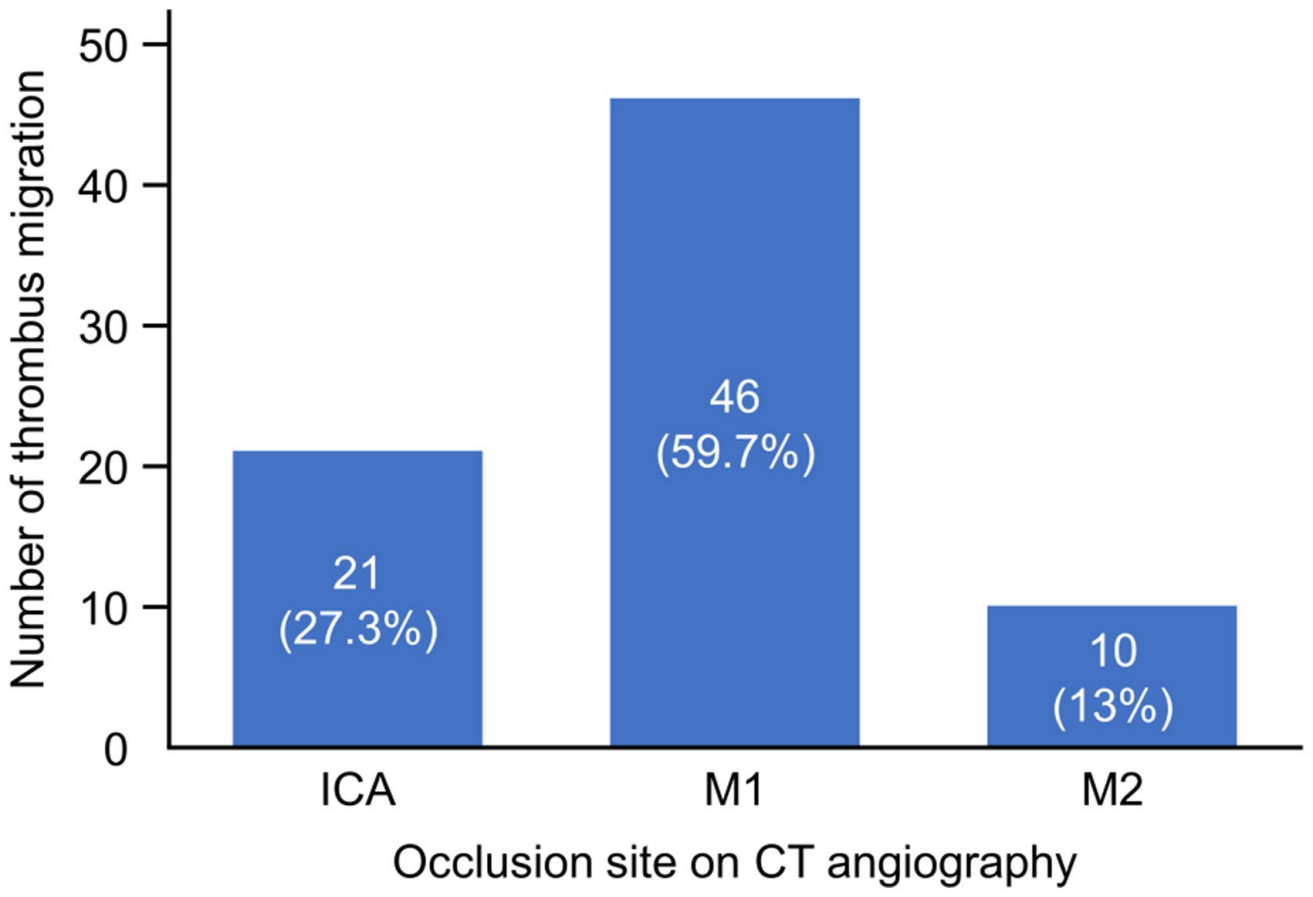
Thrombus migration across different occlusion sites on CT angiography. The graph displays the number and percentage of thrombus migration cases among patients with different occlusion sites identified on CT angiography. The x-axis represents the occlusion sites (ICA, M1, and M2), while the y-axis shows the number of thrombus migration cases. ICA, internal carotid artery; M1, M1 segment of middle cerebral artery; M2, M2 segment of middle cerebral artery.

TM occurred in 77 patients (22.1%), while thrombus growth was documented in 10 patients (2.9%). Among patients with TM, the most common initial occlusion site on CT angiography was the M1 segment (46 patients, 59.7%), followed by the ICA (21 patients, 27.3%) and the M2 segment (10 patients, 13.0%) (Figure 2). The most frequent migration patterns were from M1 to M2 segments (19 patients) and from ICA to M1 segments (13 patients). No cases of migration from M2 segments to more distal branches were observed in this EVT-treated population.

### Clinical and imaging factors associated with thrombus migration

**Figure 3:**
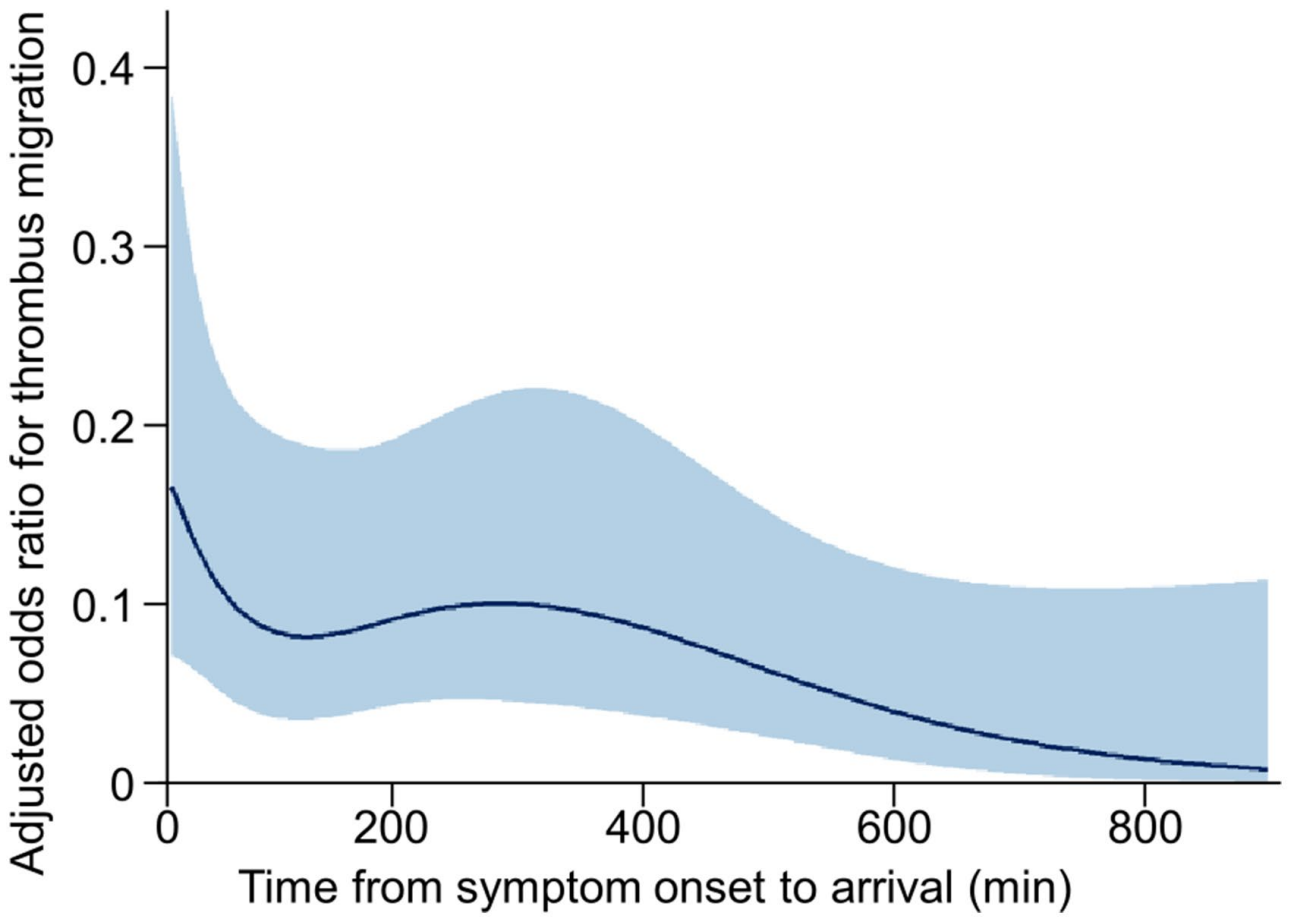
Restricted cubic spline curves of the risk for the thrombus migration according to the onset-to-arrival time. The plot illustrates the association between onset-to-arrival time and the likelihood of thrombus migration using a logistic regression model with restricted cubic splines (4 knots). The model was adjusted for age, sex, hyperdense artery sign, systolic blood pressure, diastolic blood pressure, and intravenous thrombolysis. The blue curve represents the adjusted odds ratio, and the shaded area indicates the 95% confidence interval. Odds ratios were derived by exponentiating the predicted log-odds from the spline model.

The results of univariate and multivariable logistic regression analyses for factors associated with TM are presented in Table 2. In univariate analyses, diastolic blood pressure, onset-to-arrival time per 60 min, and HAS were significantly associated with TM (*p* < 0.05), while age and systolic blood pressure showed marginal associations (*p* < 0.1). CBS, collateral status, and IVT showed no significant association with TM. Variables with a *p*-value of < 0.1 from the univariate analysis were included in the multivariable model, along with IVT based on the previous literature (3). In the multivariable analysis, HAS emerged as the strongest independent predictor of TM (adjusted odds ratio [OR], 4.68; 95% CI, 2.62–8.34), demonstrating the highest variable importance score. Diastolic blood pressure (adjusted OR, 0.98; 95% CI, 0.96–1.00) and onset-to-arrival time per 60 min (adjusted OR, 0.87; 95% CI, 0.79–0.97) also remained independently associated with TM, ranking second and third in variable importance, respectively. A restricted cubic spline analysis revealed that the odds of TM decreased with longer onset-to-arrival time (Figure 3). IVT did not show a significant association with TM in the multivariable model.

**Table 2:** Univariate and multivariable analyses for prediction of thrombus migration.

Variables	cOR (95% CI)	*p* value	aOR (95% CI)	*p* value
Age	1.02 (1–1.05)	0.074	1.03 (1–1.05)	0.058
Male	1.19 (0.72–1.99)	0.497		
Hypertension	1.01 (0.61–1.7)	0.958		
Diabetes mellitus	0.82 (0.47–1.43)	0.478		
Dyslipidemia	1.26 (0.76–2.1)	0.367		
Smoking history	1.05 (0.58–1.91)	0.873		
Prior stroke	1.6 (0.87–2.94)	0.128		
Atrial fibrillation	1.47 (0.87–2.49)	0.15		
Coronary artery disease	1.37 (0.68–2.74)	0.374		
Prior antiplatelet use	1.41 (0.81–2.45)	0.229		
Prior OAC use	1.18 (0.62–2.26)	0.61		
SBP	0.99 (0.98–1.00)	0.053	1.00 (0.99–1.01)	0.771
DBP	0.98 (0.97–0.99)	0.006	0.98 (0.96–1)	0.022
Blood glucose	1.00 (0.99–1.00)	0.461		
Baseline NIHSS	1.01 (0.97–1.06)	0.562		
TOAST classification				
LAA	1			
CE	1.08 (0.56–2.07)	0.827		
ODE	0.67 (0.17–2.63)	0.571		
UDE	0.97 (0.39–2.4)	0.953		
Onset-to-arrival (/60 min)	0.87 (0.8–0.96)	0.005	0.87 (0.79–0.97)	0.012
Arrival-to-image (/60 min)	0.73 (0.34–1.57)	0.423		
Arrival-to-IVT (/60 min)	0.8 (0.42–1.54)	0.509		
Image-to-puncture (/60 min)	0.98 (0.59–1.64)	0.952		
Puncture-to-reperfusion (/60 min)	0.81 (0.51–1.29)	0.369		
ASPECTS	0.99 (0.86–1.15)	0.915		
Hyperdense artery sign	4.21 (2.42–7.31)	< 0.001	4.68 (2.62–8.34)	<0.001
CBS	0.91 (0.82–1.03)	0.13		
Good collateral on CTA	0.8 (0.48–1.34)	0.388		
Target occlusion on CTA				
ICA	1			
M1	0.97 (0.54–1.75)	0.916		
M2	0.71 (0.31–1.66)	0.433		
Extracranial ICA occlusion	1.25 (0.54–2.92)	0.606		
IVT	1.38 (0.83–2.3)	0.216	1.09 (0.6–1.98)	0.789

cOR, common odds ratio; CI, confidence interval; aOR, adjusted odds ratio; OAC, oral anticoagulants; SBP, systolic blood pressure; DBP, diastolic blood pressure; NIHSS, National Institutes of Health Stroke Scale; mRS, modified Rankin Scale; TOAST, Trial of org 10,172 in acute stroke treatment; LAA, large artery atherosclerosis; CE, cardioembolism; ODE, other determined etiology; UDE, undetermined etiology; IVT, intravenous thrombolysis; ASPECTS, Alberta Stroke Program Early CT Score; CBS, clot burden score; CTA, computed tomography angiography; DSA, digital subtraction angiography; ICA, internal carotid artery.

### Clinical and radiological outcomes according to thrombus migration

**Figure 4:**
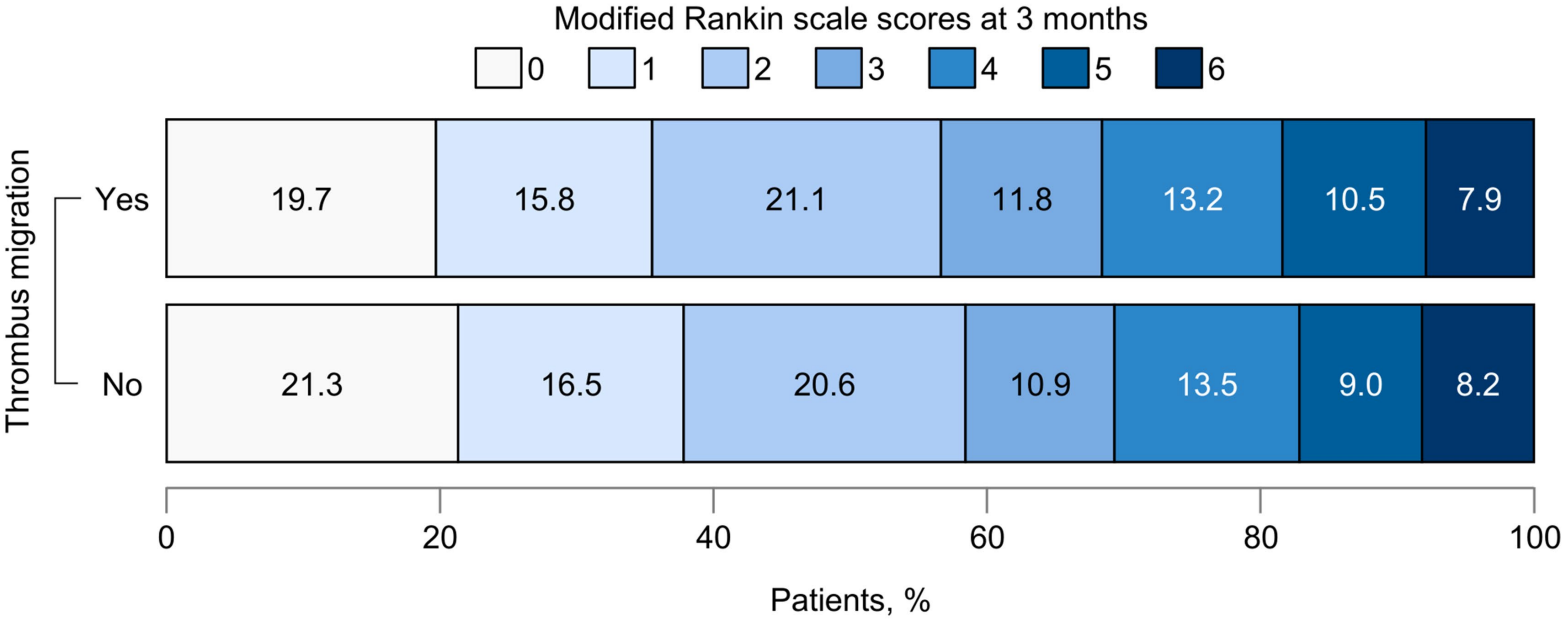
Functional outcomes at 3 months according to thrombus migration. The graph illustrates the distribution of functional outcomes at 3 months between patients with and without thrombus migration. Each bar represents one group, with segments corresponding to modified Rankin Scale scores from 0 (no symptoms) to 6 (death). The width of each segment indicates the proportion of patients within that score category.

The NIHSS score at 24 h post-procedure was lower in the TM group compared with the no-TM group (6 vs. 8), with a correspondingly higher delta NIHSS in the TM group [7 (IQR, 2–11) vs. 5 (IQR, 1–9); *p* = 0.05]. ENI was more frequent in the TM group (67.1% vs. 60.2%), though this difference was not statistically significant (*p* = 0.334). The median mRS score at 3 months was 2 (IQR, 1–4) in both groups (Figure 4). Successful reperfusion was achieved in 74 patients (96.1%) in the TM group and 249 patients (91.9%) in the no-TM group. FPE was more frequent in the TM group than in the no-TM group (29.9% vs. 20.3%, *p* = 0.105). Regarding hemorrhagic complications, parenchymal hemorrhage was more common in the TM group (13.0% vs. 5.2%), while the incidence of symptomatic ICH was similar between groups (Table 3).

**Table 3:** Clinical and radiological outcomes according to thrombus migration.

Outcome variables	No TM (*n* = 271)	TM (*n* = 77)	*p* value
Clinical outcomes			
Puncture-to-reperfusion, median (IQR)	36 (24–60)	34 (25–52)	0.514
Delta NIHSS, median (IQR) (*N* = 343)	5 (1–9)	7 (2–11)	0.05
ENI (*N* = 343)	160 (60.2)	51 (67.1)	0.334
mRS at 3 months, median (IQR) (*N* = 344)	2 (1–4)	2 (1–4)	0.74
Good outcome at 3 months (*N* = 344)	156 (58.4)	43 (56.6)	0.876
Radiological outcomes			
First pass effect	55 (20.3)	23 (29.9)	0.105
Modified first pass effect	85 (31.4)	30 (39.0)	0.266
Successful reperfusion (mTICI 2b–3)	249 (91.9)	74 (96.1)	0.31
Complete reperfusion (mTICI 3)	140 (51.7)	41 (53.2)	0.907
Distal embolization	91 (33.6)	28 (36.4)	0.75
Symptomatic ICH	9 (3.3)	1 (1.3)	0.698
Parenchymal hemorrhage	14 (5.2)	10 (13.0)	0.033
Subarachnoid hemorrhage	3 (1.1)	1 (1.3)	> 0.999

TM, thrombus migration; NIHSS, National Institutes of Health Stroke Scale; ENI, early neurological improvement; mRS, modified Rankin Scale; IQR, interquartile range; mTICI, modified Thrombolysis In Cerebral Infarction; ICH, intracranial hemorrhage.

### Effects of thrombus migration on clinical and radiological outcomes

TM was not significantly associated with clinical outcomes, including ENI (OR 1.35; 95% CI 0.79–2.31) and good outcome at 3 months (OR 0.93; 95% CI 0.55–1.55) in the univariate analysis. The effects of TM on clinical outcomes remained non-significant in multivariable analyses after adjusting for relevant prognostic variables (Table 4). Among radiological outcomes, TM was independently associated only with parenchymal hemorrhage in the multivariable analysis (adjusted OR, 2.89; 95% CI, 1.03–8.09). Additional factors independently associated with parenchymal hemorrhage included blood glucose (adjusted OR, 1.01; 95% CI, 1.00–1.02), ASPECTS (adjusted OR, 0.64; 95% CI, 0.5–0.82), and IVT (adjusted OR, 0.26; 95% CI, 0.08–0.85). TM showed a tendency toward association with FPE in the univariate analysis (OR, 1.67; 95% CI, 0.95–2.96), but it was not an independent predictor of FPE in the multivariable analysis (adjusted OR, 1.67; 95% CI, 0.9–3.08). No other radiological outcomes demonstrated a statistically significant relationship with TM in either univariate or multivariable analyses (Table 4).

**Table 4:** Association of thrombus migration with clinical and radiological outcomes in univariate and multivariable analyses.

Outcome variables	cOR (95% CI)	*p* value	aOR (95% CI)	*p* value
ENI	1.35 (0.79–2.31)	0.272	1.09 (0.54–2.2)	0.805
Good outcome at 3 months	0.93 (0.55–1.55)	0.773	0.8 (0.4–1.61)	0.539
First pass effect	1.67 (0.95–2.96)	0.077	1.67 (0.9–3.08)	0.103
Modified first pass effect	1.4 (0.83–2.36)	0.212	1.21 (0.68–2.17)	0.512
Successful reperfusion (mTICI 2b–3)	2.18 (0.63–7.48)	0.216	1.14 (0.27–4.8)	0.857
Complete reperfusion (mTICI 3)	1.07 (0.64–1.77)	0.806	0.75 (0.42–1.35)	0.339
Distal embolization	1.13 (0.67–1.92)	0.65	1.2 (0.68–2.09)	0.531
Symptomatic ICH	0.38 (0.05–3.07)	0.366	0.64 (0.07–5.99)	0.695
Parenchymal hemorrhage	2.74 (1.17–6.44)	0.021	2.89 (1.03–8.09)	0.043
Subarachnoid hemorrhage	1.18 (0.12–11.46)	0.889	0.64 (0.07–5.99)	0.695

cOR, common odds ratio; CI, confidence interval; aOR, adjusted odds ratio; ENI, early neurological improvement; mTICI, modified Thrombolysis In Cerebral Infarction; ICH, intracranial hemorrhage.

## Discussion

In this study, 22.1% of patients undergoing EVT developed TM. TM was significantly associated with HAS, diastolic blood pressure, and shorter onset-to-arrival time, but not with IVT. Patients with TM showed greater improvement in NIHSS scores and higher rates of ENI after EVT, though this did not affect 3-month functional outcomes. While TM was linked to slightly higher rates of FPE, successful reperfusion rates remained similar between groups. However, patients with TM had significantly higher rates of parenchymal hemorrhage.

Our data suggest that thrombus composition, as indicated by HAS, may be a strong predictor of TM. HAS on non-contrast CT represents erythrocyte-rich clots (20, 21) and correlates with better recanalization response to reperfusion therapy (22–24). Importantly, erythrocyte-rich clots exhibit relatively less friction against vessel walls compared with fibrin-rich clots (25). This reduced friction may enable thrombi with HAS to migrate more readily to distal arteries while also demonstrating better response to both physiologic and pharmacologic thrombolysis. This mechanism is supported by previous studies showing that TM occurs more frequently with erythrocyte-rich thrombi, as confirmed by histological examination in patients with acute ischemic stroke (26, 27). These findings align with our observations. As HAS is an effective imaging biomarker of thrombus composition that is relatively easy to assess, it could be a valuable clinical tool for predicting TM and informing procedural planning strategies. Although histological validation was not available in our cohort, HAS remains a robust surrogate marker, as it reflects native clot characteristics unaffected by periprocedural interventions.

Beyond composition, we examined temporal factors influencing TM. Few studies have explored the relationship between time parameters and TM. One previous study found that longer intervals between initial CT angiography and DSA were associated with TM, possibly due to the time needed for thrombolysis after intravenous therapy or delayed detection of migration itself (28). Conversely, our study found that the TM group had shorter onset-to-arrival times (median 75 vs. 135 min), with shorter times independently associated with increased TM risk. This finding aligns with previous reports of shorter onset-to-imaging or puncture times in TM-positive cases (3, 7, 28).

This temporal relationship can be explained by thrombus maturation dynamics. Fresh thrombi have a core-shell architecture with a fibrin/platelet-rich outer shell surrounding an erythrocyte-rich core (29, 30). Early after occlusion, the erythrocyte-rich core maintains greater deformability, making the thrombus more susceptible to migration. Over time, thrombus composition shifts toward increased fibrin content throughout, particularly reinforcing the outer shell and reducing erythrocyte proportion in the core. This structural evolution increases stability and rigidity, thereby decreasing migration tendency. Additionally, shorter onset-to-arrival times correlate with an increased use of IVT, which may promote TM through partial thrombolysis of the vulnerable erythrocyte-rich core.

Our results revealed that lower diastolic blood pressure was independently associated with an increased risk of TM. Lower diastolic blood pressure has been related to various cardiac pathologies, including impaired coronary perfusion and myocardial injury, leading to coronary artery disease and heart failure (31–33). These cardiac conditions may create a prothrombotic environment that facilitates intracardiac thrombus formation, as well as predisposition to atrial fibrillation. Consistent with these findings, previous studies have reported that lower diastolic blood pressure was associated with cardioembolic stroke, in which thrombi are typically less adherent to vessel walls compared to the *in situ* thrombi from atherosclerotic disease (34, 35). Moreover, lower diastolic blood pressure may reflect reduced peripheral vascular resistance or diminished distal perfusion pressure, potentially altering hemodynamic forces at the thrombus-vessel interface and promoting thrombus dislodgement (36). This novel association suggests the need for further research into the hemodynamic mechanisms underlying TM and may have important implications for blood pressure management strategies during the hyperacute phase of ischemic stroke before reperfusion therapy.

Several factors previously reported to be associated with TM, including IVT, collateral circulation, and CBS, showed no significant association with TM in our study. Previous studies have shown conflicting results regarding the association between IVT and TM, with some reporting a positive association (3, 7, 28, 37), while others found no relationship (38). Contrary to expectations, our analysis did not demonstrate an independent association between IVT and TM adjustment for confounders. This discrepancy from previous studies may be attributable to two temporal factors. First, our cohort had markedly shorter image-to-puncture times compared to prior studies, which closely approximated the time from IVT administration to puncture. Consequently, TM assessment on DSA may have occurred before IVT could exert its full therapeutic effect, potentially underestimating its influence on TM. Second, few studies investigating the relationship between IVT and TM have reported onset-to-arrival times in the IVT versus no-IVT groups (3, 28). In our cohort, patients who received IVT arrived much earlier than those who did not [median 66 vs. 224 min]. As thrombus characteristics change over time, this difference suggests that intrinsic clot characteristics, rather than IVT itself, may influence TM occurrence.

The absence of associations with collateral circulation status and CBS provides additional insights into TM mechanisms. These findings suggest that hemodynamic factors and clot size may be less important than intrinsic thrombus composition in determining migration likelihood.

In our study, patients with TM showed greater NIHSS improvement at 24 h and trends toward higher rates of ENI and first-pass effect. However, these early advantages did not translate into significant differences in the 3-month functional outcomes between groups. The presence of TM may indicate a more fragile or mobile thrombus composition that facilitates clot retrieval, potentially explaining the improved early neurological recovery. By definition, TM leads to more distal occlusion sites, which can alter EVT strategy, such as the use of smaller devices or even the decision to attempt EVT in very distal lesions. Nevertheless, successful reperfusion (mTICI 2b–3) was comparable between groups, indicating that EVT effectiveness was not diminished by distal migration. Previous research on TM’s clinical impact has yielded contradictory findings. Some studies report better functional outcomes with TM despite lower complete reperfusion rates (3, 7), while others found reduced successful reperfusion and consequently diminished neurological improvement in patients with TM (38). This paradox may reflect TM’s dual nature. On the one hand, TM can hinder recanalization when thrombi migrate to distal, less accessible vessels, potentially worsening outcomes. Conversely, distal migration may relieve proximal obstruction and enhance collateral perfusion, contributing to early symptom improvement. In our cohort, TM did not compromise reperfusion success and appeared to positively influence early neurological outcomes, possibly through improved distal perfusion.

Notably, TM was significantly associated with higher rates of parenchymal hemorrhage (13.0% vs. 5.2%). The underlying mechanism remains unclear. TM may not directly cause hemorrhage but rather indicate thrombus characteristics that predispose to bleeding complications. Erythrocyte-rich thrombi, which are more migration-prone as shown by our HAS findings, may respond more aggressively to thrombolytic therapy, increasing hemorrhage risk. Alternatively, distal migration may contribute to hemorrhagic complications when thrombi migrate to smaller vessels with compromised blood–brain barrier integrity. Subsequent reperfusion of these vulnerable territories could precipitate hemorrhagic transformation. Additionally, patients with TM had shorter onset-to-arrival times and may have received higher IVT during the early period when hemorrhage risk was elevated. Given the increased hemorrhage risk associated with TM, enhanced monitoring protocols may be warranted for patients presenting with predictive factors such as HAS and early onset.

Despite the higher frequency of PH in the TM group, the incidence of symptomatic ICH was similar between groups. In our study, symptomatic ICH was defined as any ICH, including SAH, that was accompanied by clinical worsening, whereas PH represents only a subtype of intracerebral hemorrhage. Accordingly, PH and symptomatic ICH do not necessarily coincide, and many PH cases may not result in significant neurological deterioration. Furthermore, distal migration in the TM group may have led to smaller or non-eloquent hemorrhages, which could also explain the absence of a higher symptomatic ICH rate despite the increased occurrence of PH.

In this study, 22.1% of patients who underwent EVT developed TM, a rate consistent with previous reports ranging from 15 to 25% (3, 8, 38). TM was most commonly observed in patients with initial ICA and MCA M1 segment occlusions, with the most frequent migration patterns being M1 to M2 (19 patients) and ICA to M1 (13 patients). The absence of migration from M2 segments to more distal branches in our EVT-treated cohort likely reflects our inclusion criteria, as patients with very distal migrations may not have met indications for mechanical thrombectomy. This selection bias inherent to EVT studies may underestimate the true incidence of distal TM in the broader stroke population. Additionally, our findings may not be generalizable to patients managed with medical therapy alone, where different migration patterns might occur.

There are several limitations in this study. First, we cannot exclude the possibility of hidden confounders because of the retrospective study design. However, we attempted to include all possible variables to find factors that were independently associated with TM. Second, because our study only included patients who underwent EVT, we could not evaluate patients who did not undergo EVT in case of TM to the far distal artery. However, it is inconclusive whether EVT is advisable for medium or distal arteries because the smaller the occluded vessel, the smaller the infarct size, the smaller the sequelae, and the higher the risk of periprocedural complications. Our study showed that TM did not adversely affect the outcome of patients undergoing EVT. Third, we evaluated TM or HAS by visual assessment without measuring migration distances or Hounsfield units. These aspects may be subject to some error. However, our study is significant in that we have assessed TM in a practical way and identified radiologic predictors for TM that can be applied in practice. Fourth, because we defined TM as any distal movement, our classification may have included both clinically meaningful migrations and subtle positional changes. This challenge is especially relevant for intrasegmental migration, where interpretation is more difficult. Developing a standardized grading system for TM severity could improve comparability across future studies. Fifth, our study did not collect data on patient transfer status, which could potentially influence TM patterns and time-related variables. Finally, we did not assess thrombus perviousness on CT angiography or perform histological analysis of retrieved thrombi, both of which could have provided additional insights into thrombus composition and its relationship to migration patterns.

In this study, 22.1% of patients with intracranial large vessel occlusion developed TM during EVT. HAS, lower diastolic blood pressure, and shorter onset-to-arrival time were independently associated with TM occurrence, suggesting that thrombus composition and maturation play a key role in migration susceptibility. While TM was associated with slightly better neurological improvement and higher rates of parenchymal hemorrhage, overall procedural success and 3-month functional outcomes were comparable between the TM and non-TM groups. These findings indicate that TM has minimal impact on EVT effectiveness and should not alter treatment decisions for patients with large vessel occlusion.

## Data Availability

The study data are available from the corresponding author upon reasonable request and with the permission of all contributing authors. Requests to access these datasets should be directed to Y-WK, yw.kim23@gmail.com.

## Glossary

TM: thrombus migration
EVT: endovascular thrombectomy
ICA: internal carotid artery
M1: M1 segment of middle cerebral artery
M2: M2 segment of middle cerebral artery
NIHSS: National Institutes of Health Stroke Scale
IVT: intravenous thrombolysis
MCA: middle cerebral artery
CT: computed tomography
TOAST: Trial of Org 10,172 in Acute Stroke Treatment
ASPECTS: Alberta Stroke Program Early CT Score
HAS: hyperdense artery sign
CBS: clot burden score
DSA: digital subtraction angiography
ENI: early neurological improvement
mRS: modified Rankin Scale
mTICI: modified Thrombolysis in Cerebral Infarction
FPE: first pass effect
ICH: Intracranial hemorrhage
ECASS: European Cooperative Acute Stroke Study
IQR: interquartile range
OR: adjusted odds ratio
